# Gastric Cancer Heterogeneity and Clinical Outcomes

**DOI:** 10.1177/1533033820935477

**Published:** 2020-08-17

**Authors:** Rachel E. Sexton, Mohammed Najeeb Al Hallak, Md. Hafiz Uddin, Maria Diab, Asfar S. Azmi

**Affiliations:** 1 Department of Oncology, 12267 Wayne State University School of Medicine, Detroit, MI, USA

**Keywords:** gastric cancer, oncomine, classification, microRNA, differential gene expression

## Abstract

Gastric adenocarcinoma is a highly aggressive disease with poor overall survival. The aggressive nature of this disease is in part due to the high intra and inter tumoral heterogeneity and also due to the late diagnosis at presentation. Once progression occurs, treatment is more difficult due to the adaptation of tumors, which acquires resistance to commonly used chemotherapeutics. In this report, using publicly available data sets and pathway analysis, we highlight the vast heterogeneity of gastric cancer by investigating genes found to be significantly perturbed. We found several upregulated genes in the diffuse gastric cancer subtypes share similarity to gastric cancer as a whole which can be explained by the increase in this subtype of gastric cancer throughout the world. We report significant downregulation of genes that are underrepresented within the literature, such as *ADH7*, *GCNT2*, and *LIF1*, while other genes have not been explored within gastric cancer to the best of our knowledge such as *METTL7A*, *MAL*, *CWD43*, and *SLC2A12*. We identified gender to be another heterogeneous component of this disease and suggested targeted treatment strategies specific to this heterogeneity. In this study, we provide an in-depth exploration of the molecular landscape of gastric cancer in order to shed light onto novel areas of gastric cancer research and explore potential new therapeutic targets.

## Introduction

Gastric cancer (GC) persists as a worldwide public health crisis. According to the American Cancer Society, the 5-year survival rate of GC remains at 25% worldwide and 31% within the United States.^1^ These survival statistics have increased overall since the 1980s when the 5-year survival rate for stage II disease was below 30% and near 0% for stage IIIB and higher.^1^ With the development of chemotherapies such as platinums and taxanes, survival beyond stage II increased steadily to 31%. Although chemotherapies improved overall survival, this is not as dramatic as that in other solid malignancies such as prostate or breast. Furthermore, even with the identification of molecular targets, such as BRCA mutations and HER2 amplifications, clinical success with available therapies has been minimal.^2,3^ A recent clinical trial with olaparib, a poly ADP ribose polymerase inhibitor, showed little efficacy compared to standard of care.^4^ Although a subset of gastric disease has HER2 amplification, monoclonal antibodies against HER2 have demonstrated very limited success in GC, unlike the response seen in HER2 positive breast cancer.^5^ It is clear that more work is needed to elucidate the underlying molecular drivers and resistance mechanisms in GC.

Gastric cancer is classified mainly using either the Lauren classification or the World Health Organization (WHO) criteria. The Lauren classification compares tumors based on growth (invasion) pattern with 3 subtypes: intestinal (well differentiated), diffuse (poorly differentiated), and intermediate (mixed).^6,7^ The majority of patients outside US with GC are younger (<60years old) and have the poorly differentiated (diffuse) subtype, which is located within the distal portion of the stomach, characterized by poor cellular differentiation and high intratumor heterogeneity. This subtype has poorer outcomes due to its widespread infiltration and invasive nature of the disease.^7,8^ Conversely, within the United States, the pathology of GC is similar to that of malignancies found within the gastroesophageal junction.^8^ Older patients are primarily impacted and the disease is commonly well differentiated (intestinal). The well-differentiated subtype is found in the cardia or lower region of the stomach with well-defined glandular structures and growth pattern. The WHO designation for GC was created in 2010 and expands vastly on the Lauren classification.^6,7^ There are 5 subtypes: tubular adenocarcinoma, papillary adenocarcinoma, mucinous adenocarcinoma, poorly cohesive (Signet ring cell carcinoma), and mixed carcinoma.^6,7^ Similarities exist between the Lauren and WHO classifications. Signet-ring cell carcinoma (comparable to poorly differentiated GC) is steadily increasing in incidence within the United States and around the world.^9^ This increase is attributed to (1) eradication efforts of Helicobacter *pylori*, a pathogen known to induce intestinal type GC, (2) increases in genetic predisposition to genes such as E-cadherin (*CDH1)* hypermethylation, and (3) less screening and detection due to the “low risk” population within the United States compared to other regions such as Japan.^10^

Here we aim to analyze the molecular signatures as well as differences between Lauren classified GCs. We also aim to understand the molecular differences between male and female patients with GC. We chose to look solely at Lauren classified cancers within this article due to its established use within the medical community as well as its availability and relevance within publicly available data sets. Our overarching goal is to identify and dissect some of the heterogeneous aspects of GC that are commonly overlooked within the literature.

## Methods

### Oncomine Database Search

Oncomine (Compendia Bioscience) was used for analysis and visualization. Three separate data sets were used to explore the up- and downregulation of Lauren subtypes of GC: *Chen Gastric (Mol Biol Cell, 2003, mRNA), DErrico Gastric (European Journal Dataset2, 2009, mRNA)*, and *Cho Gastric (Clinical Cancer Research, 2011, mRNA)*. For the nonsubtyped GC analysis, we have used 3 separate data sets: *Cui Gastric (Nucleic Acids Research, 2011, mRNA)*, *Wang Gastric (Medical Oncology, 2010, mRNA)*, and *Cho Gastric (Clinical Cancer Research, 2011, mRNA)*. To find highly ranked genes, we selected our subtype of interest (or GC) compared to normal and assessed upregulated or downregulated genes. We averaged the fold changes for genes in the individual analyses and have used the computed *P* values provided by the Oncomine software.

### Kyoto Encyclopedia of Genes and Genomes Pathway Analysis

To identify pathways involved in the genes found to be upregulated or downregulated from our Oncomine analysis, we utilized the Kyoto Encyclopedia of Genes and Genomes.

### MiRWalk Database Analysis

MiRWalk Database (University of Heidelberg) was used for analysis of gene–microRNAs (miRNA) interactions.^11^

### Drug–Gene Interaction Analysis

DGIdb database was used to identify druggable targets within our genes found to be differentially expressed.^12^

### Protein Database

The Human Protein Atlas (available from http://www.proteinatlas.org) was used to identify survival curves in stomach cancer with the following proteins: *CWD43* (Stage I-IV Survival curves https://www.proteinatlas.org/ENSG00000109182-CWH43/pathology/stomach+cancer), *METLL7A* (Stage I-IV https://www.proteinatlas.org/ENSG00000185432-METTL7A/pathology/stomach+cancer), *SLC2A12* (Stage I-IV https://www.proteinatlas.org/ENSG00000146411-SLC2A12/pathology/stomach+cancer), *MAL* (Stage I-IV https://www.proteinatlas.org/ENSG00000172005-MAL/pathology/stomach+cancer), DMRT1 (Stage I-IV https://www.proteinatlas.org/ENSG00000137090-DMRT1/pathology/stomach+cancer). All are available from v19.proteinatlas.org.

### Protein–Protein Interaction Networks

STRING 3.0 Database was used to identify protein–protein interactions for the following genes: *CWH43*, *METLL7A*, *SLC2A12*, *MAL*, *BTD*, *CAPN9*, *ADAM17*, *EPB41*, *TOM1L1*, and *DMRT1*.^13^

### GEO Database Analysis

The data discussed within this publication have been previously deposited in NCBI’s Gene Expression Omnibus and are accessible through GEO Series accession number GSE118916 (https://www.ncbi.nlm.nih.gov/geo/query/acc.cgi?acc=GSE118916.

### Statistics

Oncomine software and Human Protein Atlas provided Statistics.

### Ethical Approval

The data are not obtained from patients and does not require institutional review board approval.

## Results

### Genetic Analysis of Upregulated GC Genes

Within the literature, various genetic aberrations have been proposed that can serve as prognostic or therapeutic markers including *SOX17* hypermethylation, *BCL2*, transforming growth factor beta (TGF*-β)*, vascular endothelial growth factor (*VEGF*)*/R*, and *HER2.* ^14-18^ Many of these proposed markers are studied extensively and do not serve as ideal targets due to their limited clinical utility as either drug targets or predictors of therapeutic response. Some examples of this include less successful attempts to target *HER2* with monoclonal antibodies and the use of *TGF-β* inhibitors, which although promising, have proven to be highly toxic.^19,20^ Additionally, these targets have demonstrated limited clinical utility due to the crosstalk between *TGF-β* and other signaling pathways such as *RAS*, a known nontargetable protein.^21,22^ While *VEGF* inhibitors are used as a therapeutic modality in GC, they do not improve overall survival.^23^ An in-depth investigation of the molecular mechanisms are urgently and investigations need to be distinct from the commonly studied and clinically intractable targets. Although this is the case, discrepancies exist within the literature as some groups look at the molecular composition of GC as a whole while others focus on differences within the Lauren classification system.

Using the Oncomine database, we have found significant upregulation in several under-studied genes in all GCs including *COL3A1*, *COL5A2*, *SPON2*, and *CDH11* (Table 1). We also have confirmed the upregulated status of many of the genes found within the literature that are somewhat well known such as *INHBA*, a gene associated with poor overall outcomes,^24^ but are still understudied. Claudin 1 (*CLDN1)* has been found to be highly expressed in GC and is a poor predictive disease marker by mediating tumor necrosis factor-α induced cell migration, enhancement of proliferation, and metastasis while *SULF1* has been found to be significantly hypomethylated causing significant downregulated protein expression.^25-28^ This *SULF1* downregulation may be indicative of a posttranslational modification, feedback loop, or degradation event via protein–protein interactions but is still unclear. Not surprisingly, a significant underrepresentation was noted when comparing publications related to these genes (over 100 publications) to the commonly studied genes such as *MAPK*, *PI3K*, and *TP53* (over 3000 total publications).

**Table 1.** Top Upregulated Genes Found in Gastric Cancer Cohort via Oncomine Database.^a^

Gene name	Fold change diffuse vs normal (average)	*P* value	Publications found
*INHBA*	13.253	5.49E-7	12
*COL1A2*	4.890	9.49E-12	55
*CLDN1*	8.674	6.64E-6	19
*CDH11*	2.638	1.17E-10	6
*COL3A1*	2.581	2.41E-6	6
*COL5A2*	2.870	2.89E-6	6
*COL1A1*	4.543	2.99E-6	11
*TIMP1*	3.190	3.83E-6	40
*SULF1*	5.094	4.65E-6	9
*SPON2*	2.436	6.44E-10	3

^a^ *P* values were calculated using Oncomine software.

### Genetic Analysis of Upregulated GC Genes Using Lauren Type Classified GCs

We stratified the data sets based on the respective Lauren distinguished subtype and have highlighted the vast heterogenetic molecular landscape within the poorly differentiated (diffuse), well differentiated (intestinal), and mixed GC subtypes (Table 2). Poorly differentiated GC shares many similarities with GC overall including perturbations in various collagen-transcribing genes, stimulation of PI3K/AKT signaling, and perturbations in cellular structural components. This is a dominant subtype throughout the world for reasons we have previously mentioned. Due to the overabundance of collagen transcribing genes, we wanted to explore whether a potential genetic link exists. Literature search identified a study correlating Ehlers-Danlos syndrome (EDS), a disease caused by collagen gene perturbations, to the development of GC.^29^ Ehlers-Danlos syndrome also presents with gastrointestinal involvement such as increased rates of heartburn, which is a risk factor for developing esophageal cancer.^30,31^ Based on the location of these gastric tumors within the stomach that is, in the proximal stomach near the esophagus, and the connection between gastric and esophageal cancers, it is quite possible there may be a much stronger correlation between EDS and diffuse GC than previously thought.

**Table 2.** Top Significantly Upregulated Genes Based on Molecular Subtype of Gastric Cancer (Well Differentiated, Poorly Differentiated, Mixed Subtype) Based on Oncomine Database.^a^

Gene name	Fold change diffuse vs normal (average)	*P* value	KEGG pathway analysis	Gastric cancer subtype
*THY1*	4.681	1.61E-12	Immune component	Diffuse
*TIMP1*	3.392	1.24E-11	HIF signaling	Diffuse
*BGN*	4.782	2.38E-11	–	Diffuse
*COL1A2*	5.831	2.23E-10	PI3K/AKT, focal adhesion, ECM receptor, proteoglycans	Diffuse
*SULF1*	6.540	1.39E-9	Metabolism	Diffuse
*COL6A3*	4.225	5.85E-9	PI3K/AKT, focal adhesion, ECM receptor	Diffuse
*OLFML2B*	2.828	4.04E-8	–	Diffuse
*RAB31*	2.667	3.61E-9	Membrane trafficking	Diffuse
*THBS2*	4.484	1.18E-8	Phagosome, PI3K/AKT, focal adhesion, ECM–receptor interaction	Diffuse
*COL1A1*	6.731	1.65E-7	PI3K/AKT, focal adhesion, ECM receptor, proteoglycans	Diffuse
*TTYH3*	2.585	2.32E-23	Transporter	Intestinal
*THY1*	3.474	3.46E-21	Immune component	Intestinal
* CAD*	2.528	2.02E-8	Phenylpropanoid biosynthesis, metabolic pathways, biosynthesis of secondary metabolites	Intestinal
*UBE2C*	2.728	2.62E-20	Ubiquitin-mediated proteolysis	Intestinal
*CLDN1*	5.87	6.50E-15	Cell adhesion, tight junction	Intestinal
*PRC1*	2.883	1.34E-14	Tubulin binding protein	Intestinal
*DAZAP1*	2.166	6.80E-8	mRNA surveillance	Intestinal
*ATP11A*	2.441	7.68E-19	Metabolism, translocase	Intestinal
*DCAF13*	2.066	9.71E-8	Ribosome biogenesis	Intestinal
*MTHFD1L*	2.415	8.93E-9	One carbon metabolism	Intestinal
*COL6A3*	4.168	1.09E-7	PI3K/AKT signaling, focal adhesion, ECM–receptor interaction	Mixed
*FBN1*	3.427	1.91E-7	TGF-β signaling	Mixed
*RCC2*	1.846	1.61E-9	–	Mixed
*AHCY*	2.155	2.13E-6	Cysteine and methionine metabolism	Mixed
*TGIF1*	2.257	7.33E-9	TGF-β signaling	Mixed
*FN1*	5.193	9.43E-9	PI3K/AKT signaling, focal adhesion, ECM–receptor interaction, regulation of actin cytoskeleton, proteoglycans, and pathways in cancer	Mixed
*MYO9B*	1.231	2.24E-6	Membrane trafficking	Mixed
*VCAN*	3.572	2.60E-6	Cell adhesion molecules (CAMs)	Mixed
*LUM*	2.756	3.80E-6	Proteoglycans in cancer	Mixed
*MCM4*	2.612	8.33E-6	DNA replication, cell cycle	Mixed

Abbreviations: ECM, extracellular matrix; KEGG, Kyoto Encyclopedia of Genes and Genomes; TGF-β, transforming growth factor beta.

^a^ *P* values were calculated via Oncomine software and KEGG pathway analysis was used to analyze gene function.

We have found GC overall does not share many molecular similarities with the well-differentiated subtype of GC within the scope of our analysis. We have found only a similarity *CLDN1* expression. Claudin 1 is a gene involved in coding for the protein involved in epithelial barrier functions and is part of the claudin family. Within GC, *CLDN1* has found to be differentially expressed in GC and has been found to be upregulated in a small patient population being linked to poor survival outcomes indicative of an oncogenic function.^32^ Other groups have found claudin-1 has tumor suppressive activities and can reverse the epithelial-to-mesenchymal transition in GC cells and was found to be downregulated in intestinal type GC in a of 72 patients cohort.^33,34^ It is clear that work needs to be done in order to elucidate the role *CLDN1* plays within intestinal type gastric tumors as it has differing functions based on the literature. Many of the processes underlying intestinal GC involve alterations in metabolism and cellular crosstalk (Table 2). It is not surprising that the intestinal and diffuse GCs are distinctly different but we did find similarity with *THY1* expression both having similar fold changes. Although this gene has not been investigated in GC, it is overexpressed in the pancreatic cancer microenvironment.^35^ Further investigation may be needed as this gene may have importance in GC development.

We finally investigated the mixed subtype of GC, a subtype that is commonly overlooked within the literature (Table 2). Interestingly, mixed GC has some similarities to the diffuse subtype including PI3K/AKT signaling, a collagen transcribing gene and upregulation of cellular organizational components. Interestingly, we have found the genes perturbed within this subtype are involved in driving a number of genetic diseases such as Marfan syndrome (*FBN1)* and hypermethioninemia (*AHCY)*. Research has shown Marfan syndrome, due to aberrant *TGF-β* signaling, can induce GC development in a murine model.^36^ Hypermethioninemia, which can go undetected for years, was found to induce aggressive cancers by protecting tumors from 5-flurouracil (5-FU)-induced death, a chemotherapy commonly used to treat GC.^37,38^ It is likely the diffuse subtype is not the only subtype with a strong genetic link but the mixed subtype may have a stronger genetic component than previously thought. We hypothesize some of the genetic diversity within GC is masked when analyzed as a whole, which further supports the notion of this disease being highly heterogeneous.

### Genetic Analysis of Downregulated GC Genes

**Figure 1.**
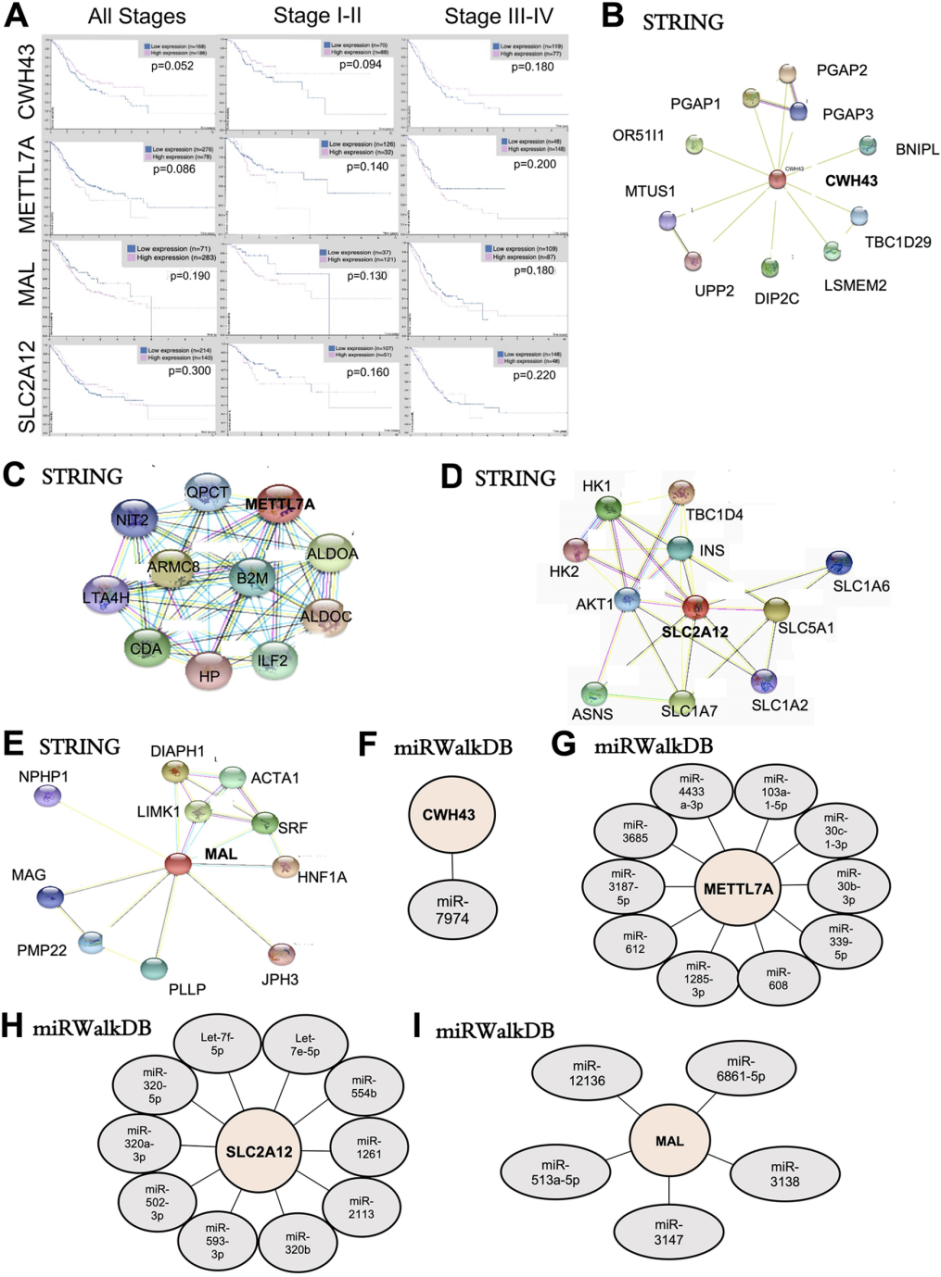
Gastric cancer is a highly heterogeneous disease. A, Survival curves taken from the human protein atlas for CWH43, METTL7A, SLC2A12, and MAL. B-D, Protein interaction networks for CWH43, METTL7A, SLC2A12, and MAL taken from the STRING Database. E-H, miRNA interaction networks found from top interactions with CWH43, METTL7A, SLC2A12, and MAL in the miRDb 3.0.

There are about twice as many published studies looking at upregulated GC genes compared to downregulated (∼500 vs 1200). The most common downregulated GC genes are influenced in part by aberrant DNA methylation.^39,40^ Other than this, much less is studied pertaining to highly significant downregulated genes in GC. Using the Oncomine database, we have found the most significant downregulated genes were *LIFR*, *RDH12*, *MSFD4*, *ATP4B*, *GHRL*, and *ADH7.* All of these are poorly represented within the literature (Table 3). We have investigated the survival outcomes of select genes from table 3 that have not been investigated in gastric cancer to the best of our knowledge. These genes include METTL7A, MAL, SLC2A12 and CWH43 (Figure 1A). We found a trend toward improved survival with upregulated *CWH43* and downregulated *METLL7A*.

**Table 3.** Top Significantly Downregulated Genes According to Oncomine Database in Gastric Cancer.^a^

Gene name	Fold change diffuse vs normal (average)	*P* value	KEGG pathway analysis
*LIFR*	−2.873	2.51E-6	Cytokine–cytokine receptor interaction, signaling for pluripotency in stem cells, JAK-STAT signaling
*CWH43*	−4.101	2.79E-9	–
*RDH12*	−4.772	1.36E-8	Retinol metabolism, metabolic pathways
*MFSD4*	−7.271	2.20E-5	–
*METTL7A*	−2.349	2.27E-5	–
*ATP4B*	−128.15	1.65E-10	Oxidative phosphorylation, metabolic pathways, gastric acid secretion
*SLC2A12*	−2.919	3.65E-10	Transporter
*GHRL*	−22.079	6.17E-8	cAMP signaling, neuroactive ligand–receptor interaction, growth hormone synthesis, secretion and action
*MAL*	−4.524	1.19E-9	–
*ADH7*	−4.774	9.47E-8	Glycolysis/gluconeogenesis, fatty acid degradation, tyrosine metabolism, retinol metabolism, chemical carcinogenesis

Abbreviation: KEGG, Kyoto Encyclopedia of Genes and Genomes.

^a^ *P* values were calculated via Oncomine software and KEGG pathway analysis was used to analyze gene function.

We have included protein interaction networks for the 4 genes we have obtained using the STRING database (Figure 1B-E). *SLC2A12* interacts with *AKT1*, a commonly studied gene of interest within GC known to contribute to chemoresistance.^41^ Although many of the interacting proteins are not as well studied as *AKT1*, various genes such as *MTUS1*, *PGAP3*, *ALDOA*, and *PMP22* have been shown within the literature to only influence GC but pancreatic cancer as well.^42-46^ It is clear that further investigation into these understudied specific genetic interaction networks are needed. We then wanted to look into whether any of these genetic aberrations or their interactor proteins were targetable. To do this we utilized the DGIdb. *METTL7A* is a methyltransferase that is located primarily in lipid droplets and is silenced via DNA methylation in thyroid cancer.^47^ There is a variety of drug interactions within the network of *METTL7A* including *CDA* (gemcitabine, cytaribine, deoxycytidine), *LTA4 H* (Kelatophan, Ubenimex, and a variety of preliminary drug compounds), *B2 M* (pembrolizumab), *QPCT* (pramipexole), ALDOA (a variety of preliminary compounds), and *HP* (Estradiol, pyridoxine). Pembrolizumab has been FDA approved for the treatment of advanced staged GC with positive PDL1 expression. *B2M* acquired mutations were found to confer resistance to pembrolizumab in other malignancies^48^ but little is known in GC. Downregulation of these genes may partially explain why there is some efficacy issues with pembrolizumab or other chemotherapies. *MAL* encodes a membrane protein within the endoplasmic reticulum (ER) of T-cells and is involved in myelin biogenesis.^49^ Drug interactions within the network include *ACTA1* (kabiramide c, latrunculin a/b, aplyronine a, and a variety of preclinical compounds), *LIMK1* (dabrafenib), *PMP22* (progesterone), and *MAG* (GSK-249320). *CWH43* is involved in cell wall biogenesis and involved in lipid remodeling.^50^ Drugs that interact with the protein network include *UPP2* (fluorouracil, brivudine). Understanding the genetic landscape of GC, gene interaction networks and how those genes respond to therapies may explain partially why this disease is highly resistant to conventional chemotherapies. However, more work is needed to understand the possible underlying resistance mechanisms within subsets of GC that would bring forward the ideal populations that benefit from conventional and commonly used therapies.

Increasing interest has been placed around small RNAs including miRNAs involvement within GC development.^51,52^ We wanted to investigate the interaction networks between these uncharacterized genes of interest (bold) and miRNAs. Using the miRWalk database, we found miRNA to interact with our genes of interest (Figure 1F-I). Many of the miRNAs are uncharacterized in GC but we did find that miRNA-612 (miR-612 a *METTL7A* interacting miRNA) induces PAX8, a tumor-suppressor, and represses FOXM1 to inhibit angiogenesis and metastasis of GC.^53^ Our lab’s work in part involves (1) studying the role of nuclear export and miRNA expression and (2) uncovering ways in which tumor suppressive miRNAs can be upregulated within the nucleus by manipulating nuclear export. Nuclear export via XPO1 has a limited role in exporting miRNA from the nucleus to the cytosol rather than its nuclear export family member XPO5, which exports the majority of cellular miRNAs.^54^ XPO1 overexpression was found to be a therapeutic target in GC and we have found blocking the protein with the small FDA approved molecule selinexor (XPOVIO) influences the expression of a subset of tumor-associated miRNAs.^55^ Furthermore, we have found via small RNA sequencing that after XPO1 inhibition with selinexor as well as the second generation inhibitor KPT-8602, miR-7977 (*CWH43* interacting miRNA) is significantly upregulated (fold change 2.22, *P* = 3.92E-23 and fold change 2.08, *P* = 5.46E-20) in the early stage diffuse gastric cell line SNU-1 suggestive of the tumor suppressive role of this miRNA. The connection between nuclear export and cancer-specific miRNAs in GC has not been investigated in depth. We are working toward not only characterizing this novel interaction but also using this information to uncover novel genes pertinent to GC growth and development.

### Genetic Analysis of Downregulated GC Genes Using Lauren Type Classified GCs

We stratified the data sets based on the respective Lauren distinguished subtype as we did previously and have highlighted the vast heterogenetic molecular landscape within the diffuse, intestinal, and mixed (Table 4) GC subtypes. All subtypes are expectedly distinct from one another within our molecular analysis. The diffuse and intestinal type GCs seem to have more prominent downregulation of metabolism related genes such as *GSTA2* and *DBT. GSTA2* is involved with chemoresistance due to the action of glutathione metabolism, an antioxidant, and this observation suggests that this subtype may be more sensitive to platinum drugs.^56^ This overall downregulation of metabolic pathways may also point to an increase in the Warburg effect. This alternative metabolic pathway has been suggested to contribute phenotypically to high rates of invasion and aggressive GCs.^57^ We also observed downregulation of *ADRB2* in the intestinal type GC (Table 4). Zhang *et al* described ADRB2 signaling as essential in GC and is likely related to stress-induced tumor induction.^58^ They suggest treating with antagonists of ARDB2 likely will provide survival benefit. This may be important to note and be beneficial for nonintestinal like GCs because there is a clear trend of significant downregulation of this gene (−2.631 fold difference).

**Table 4.** Top Significantly Downregulated Genes Based on Molecular Subtype of Gastric Cancer (Well Differentiated, Poorly Differentiated, Mixed Subtype) Based on Oncomine Database.^a^

Gene name	Fold change diffuse vs normal (average)	*P* value	KEGG pathway analysis	Gastric cancer subtype
*MT1G*	−5.518	1.43E-4	Mineral absorption	Diffuse
*MT1F*	−3.673	2.13E-10	Mineral absorption	Diffuse
*GCNT2*	−3.334	5.97E-7	Glycosphingolipid biosynthesis, metabolism	Diffuse
*SLC9A1*	−2.545	7.62E-7	Transporter	Diffuse
*PPFIBP2*	−1.975	1.50E-9	-	Diffuse
*DBT*	−2.177	6.54E-4	Valine, leucine, isoleucine degradation, propionate metabolism, metabolic pathway	Diffuse
*MT1M*	−2.712	9.03E-7	-	Diffuse
*PXMP2*	−2.745	1.72E-9	Peroxisome	Diffuse
*MT1H*	−4.660	1.13E-6	Mineral absorption	Diffuse
*GSTA2*	−5.554	2.31E-9	Glutathione metabolism, drug metabolism, platinum drug resistance, pathways in cancer, chemical carcinogenesis	Diffuse
*MAL*	−5.140	8.81e-11	Ribosome biogenesis	Intestinal
*PGA3*	−71.87	4.54e-12	Protein digestion and absorption	Intestinal
*SIDT2*	−2.590	1.99E-10	-	Intestinal
*ADRB2*	−2.631	1.03E-12	cAMP signaling, neuroactive ligand–receptor interaction	Intestinal
*BRP44 L*	−1.842	1.88E-12	Mitochondrial biogenesis	Intestinal
*SST*	−8.869	4.22E-8	cAMP signaling, neuroactive ligand–receptor interaction, gastric acid secretion, growth hormone synthesis, secretion and action	Intestinal
*GCNT2*	−3.803	2.06E-12	Glycosphingolipid biosynthesis, metabolic pathways	Intestinal
*CKMT2*	−4.205	5.37E-8	Arginine and proline metabolism, metabolic pathways	Intestinal
*RAB27A*	−2.279	2.58E-12	Membrane trafficking	Intestinal
*STK32B*	−2.238	1.56E-9	Metabolism	Intestinal
*FGA*	−9.765	3.18E-10	Membrane trafficking	Mixed
*PXMP2*	−3.044	1.75E-8	Peroxisome	Mixed
*NRXN1*	−2.424	1.90E-7	Cell adhesion molecules (CAMs)	Mixed
*GSTA2*	−5.892	1.55E-6	Glutathione metabolism, drug metabolism, platinum drug resistance, pathways in cancer, chemical carcinogenesis	Mixed
*PKIB*	−3.934	1.84E-6	-	Mixed
*POU2AF1*	−3.217	8.90E-7	-	Mixed
*SLC22A23*	−2.003	1.11E-5	Organic acid transporters	Mixed
*AQP4*	−4.677	3.84E-6	Bile secretion, vasopressin-regulated water absorption	Mixed
*MLX*	−1.492	1.39E-5	Insulin resistance, nonalcoholic fatty liver disease (NAFLD)	Mixed
*CXCL14*	−3.737	1.39E-5	Cytokine–cytokine receptor interaction, viral protein interaction, chemokine signaling pathway	Mixed

Abbreviation: KEGG, Kyoto Encyclopedia of Genes and Genomes.

^a^ *P* Values were calculated via Oncomine software and KEGG pathway analysis was used to analyze gene function.

We next assessed the molecular aberrations in the downregulated genes of mixed subtype GC (Table 4). Interestingly, we found various genes that are significantly downregulated with no pathway analysis and no real evidence of a mechanism at the protein level (Table 4). *PKIB* function has not been explored within the literature in regard to GC but has been shown to promote proliferation through PI3K/AKT pathway in breast cancer.^59^ *POU2AF1* is another gene that has not been characterized within the GC literature but has been found to be a high-risk gene in gastrointestinal stromal tumors, a type of soft tissue sarcoma and rheumatoid arthritis.^60,61^ Again, the mixed subtype is molecularly different from the intestinal and diffuse gastric subtypes based on this genetic pathway analysis with notably less involvement of metabolism related genes. Although this is expected due to its difference in subtyping, the mixed gastric subtype has a much smaller representation within the literature than the intestinal and diffuse types and it is clear that further investigation is needed. A better understanding of the diverse nature of downregulated genes in all aspects of GC is needed as a first step to identify new therapeutic options that will benefit patients with GC.

### Gastric Cancer Exhibits High Molecular Differences Between Genders

Within the United States, men and women older than 65 are at higher risk for developing GC while the male population is higher in risk for well-differentiated GC development than the female population mainly due to the protective effect of estrogen against developing *H pylori* induced gastric carcinogenesis.^62^ Females have higher incidence of poorly differentiated GCs compared to their male counterparts for reasons largely unknown. Various environmental factors play a role in disease development as a whole including obesity, smoking, drinking, and a poor diet.^63-66^ A retrospective study by Kim *et al* has shown that women not only have a higher incidence of diffuse type GC but have a worse overall prognosis as well as genetic differences compared to men including ER-b expression^67^ suggesting a hormonal component may also be a contributing factor to this subset of disease. Due to the evident gender disparities in GC, we investigated the underlying molecular differences between male and female patients by preforming GEO2R analysis on the GSE118916 data set. Our results show striking differences in differentially expressed genes between males and females.

**Figure 2.**
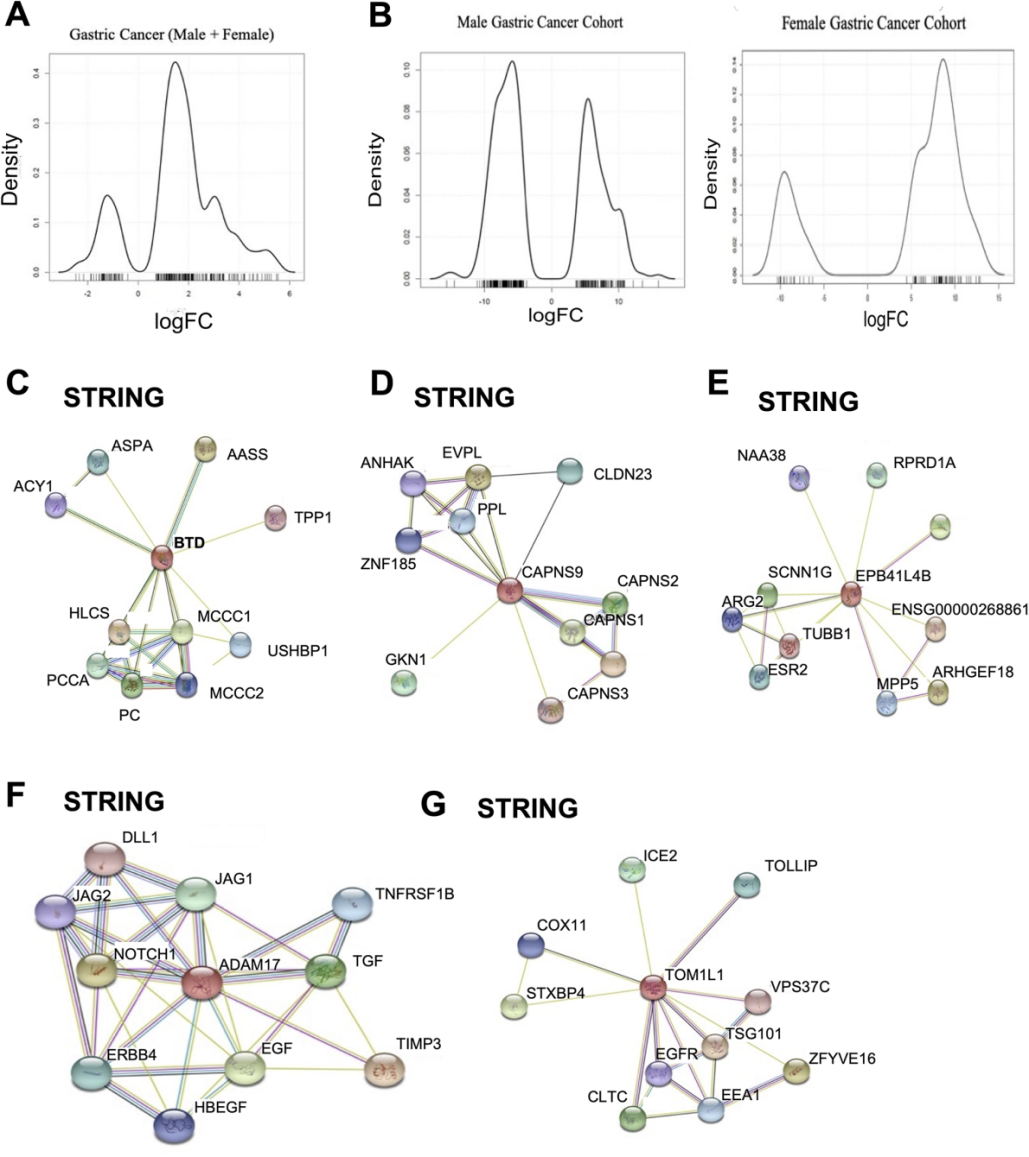
Male and female patients with gastric cancer have different molecular signatures. A, Density plots of 250 differentially expressed genes in the GSE118916 data set for all gastric cancer cases within the cohort. B, Male and female cohort density plots of the 250 differentially expressed genes in the GSE118916 data set. C-G, STRING Database interaction networks for protein networks from genes found to be differentially expressed in female gastric cancer cases within the cohort (BTD, CAPNS9, EPB41L4B, ADAM17, TOMIL1).

Overall both male and female patients with GC showed an abundance of upregulated genes (Figure 2A). After stratifying based on gender, the female patients with GC have a higher abundance of upregulated genes (oncogenic like genes) >50 genes greater than 5-fold upregulation compared to downregulated genes (Figure 2B), while male patients with GC have a greater abundance of downregulated genes (tumor suppressor like genes; Figure 2B). This trend can also be seen from just the top differentially expressed genes in the provided tables. Current treatment options for GC are somewhat limited in achieving a long-term survival benefit and we wanted to use our cohorts to identify whether there are differences in actionable targets between genders.

### Female Patients With GC Are Vastly Underrepresented Within Clinical Studies

We found no direct druggable targets (according to the DGIdb database) with the top differentially expressed genes. Therefore, we looked further into the individual protein–protein interaction networks using STRING database (Figure 2C-F). Broadening the scope of our search allowed us to find many potential druggable targets (Table 5). We narrowed the scope of our search to inhibitors/antagonist type compounds due to the substantial genes found to be upregulated. Many of the druggable targets, such as estimated glomerular filtration rate (EGFR) tyrosine kinase inhibitors (TKIs), are currently being explored in a variety of malignancies including GC. Erlotinib was investigated in a phase II clinical trial in combination with oxaliplatin/leucovorin/5-FU in metastatic GC.^68^ Lapatinib, a TKI responsible for inhibiting HER2/neu and EGFR, was tested in a phase III clinical trial (TyTAN Trial) in Asian patients with GC.^69^ There was no statistically significant difference in overall survival for Paclitaxel plus Lapatinib over Paclitaxel alone.^70^ We looked further into the patient demographics of the TyTAN trial and noticed a large underrepresentation of female patients within all arms of the study (16%-23% total female patients). Another example of this is a trial with Bortezomib, which interacts with the *ADAM17* pathway, and has been tried unsuccessfully in Phase II clinical trials in combination with paclitaxel and carboplatin in metastatic patients with GC.^71^ As with the Lapatinib trial, this one had an overrepresentation of male patients (89%) compared to female patients (11%).^71^ A common occurrence within many of the GC clinical trials is combination of new therapies with paclitaxel or some type of Taxol. We have found the female cohort to have an abundance of druggable targets interact with paclitaxel including *EPB41L4B* and *CAPN9* (Table 5) but largely this demographic is underrepresented within clinical trial studies. It is clear that based on the molecular profile of female patients with GC, this issue demands further investigation.

**Table 5.** Genes Found to Be Significantly Differentially Expressed Within the Female Cohort From the GEO Database (GSE118916).^a^

Gene name	Fold change diffuse vs normal (average)	*P* value	Drug
*FBX13*	3.192	1.09E-9	-
*DMRTA1*	2.210	2.01E-8	Testosterone, Tretinoin LY-294002
*BTD*	1.074	2.01E-8	Biotin, Hydrocortisone, Aspartic Acid, Celiponase alfa
*PFDN2*	−1.103	3.19E-9	-
*GRAMD1C*	1.713	5.33E-8	-
*CAPN9*	3.451	6.20E-8	Emricasan, Paclitaxel, Rizatriptan, Celecoxib, Idronoxil
*PBLD*	2.808	9.56E-8	-
*EPB41L4B*	2.605	9.61E-8	Paclitaxel, Vindesine, Colchicine, Docetaxel, Cabzitaxel, Erbulin mesylate, Ixabepilone, Lexibulin, Tamoxifen, Ornithine
*ADAM17*	−0.863	1.44E-7	Cetuximab, Nimotuzumab, Tesevatinib, Infliximab, Etanercept, Adalimumab, Golimumab, Hydrocortisone, Everolimus, Methotrexate, Mercaptopurine, Bortezomib, Prednisolone, Dexamethasone, Ribociclib, Nitrogacestat, Dacomitinib, Lapatinib, Erlotinib, Poziotinib, Ibrutinib, Pelitinib
*TOM1L1*	1.694	1.55E-7	Erlotinib, Afatinib, Gefitinib, Cetuximab, Lapatinib, Panitumumab, Rociletinib, Icotinib, Lacomitnib

^a^ *P* Values were calculated via the GEO Database. Druggable interactions were identified using DGIdb targets identified in protein–protein interactions from the genes listed using the String Database.

### Male Patients With GC May Benefit From Hormone Inhibiting Therapies

As we have previously mentioned, the male cohort has an opposite molecular profile compared to the female cohort with. When screening for actionable drug targets, we limited the scope of our analysis to agonists due to the substantial genetic downregulation already occurring naturally and notion that male patients with GC have an abundance of tumor suppressor like genes. In doing so, we have found direct druggable targets such as *SSTR1* and *GPT* (Table 6). *GPT* is a gene that encodes the alanine aminotransaminase 1 protein and catalyzes the reversible transamination between alanine and 2-oxoglutarate within the tricarboxylic acid (TCA) cycle to generate pyruvate (a TCA intermediate) and glutamate.^72^ Glucagon and tacrolimus interact with *GPT* but the stimulation of this gene would likely enhance glucose metabolism through the TCA cycle likely being nonbeneficial as a treatment option. Furthermore, Tacrolimus can influence the development of lymphomas.^73^ Although targeting *GPT* would not be beneficial, targeting *SSTR1* may have more benefit. Hypermethylation of *SSTR1* was found to contribute to the pathogenesis of GC by acting in a tumor suppressive manner. This hypermethylation was found to be caused by Epstein-Barr virus infection,^74^ a positive prognostic marker seen in GCs. Drugs that interact with SSTR1 include octreotide and other somatostatins. In preclinical settings, these compounds have been shown to inhibit GC growth *in vitro* and *in vivo*,^75^ and this treatment strategy may benefit male patients with GC. We have also found *PIK3C2G* to be downregulated. According to the results in our studied cohort, this gene behaves in a tumor suppressive manner rather than oncogenic, which is uncommon with other genes of the PI3K family, but *PIK3C2G* has not been functionally characterized to the best of our knowledge.

**Table 6.** Top Differentially Expressed Genes for Male Patients With Gastric Cancer in Cohort GSE118916 and Druggable Targets for Genes Were Included Using DGIdb.^a^

Gene name	Fold change diffuse vs normal (average)	*P* value	Drug
ANO7	−3.06	3.09E-12	-
LNX1	−2.304	5.57E-12	-
PIK3C2G	−4.32	6.81E-12	No agonists
SSTR1	−4.424	3.87E-11	Pasireotide, Alendronic acid, Cortistatin-14, Somatostatin, Octreotide, Octreotide-acetate
*GPT*	−1.745	4.76E-11	Glucagon, Tacrolimus
*DMRTA1*	−2.041	7.97E-11	Testosterone, Tretinoin, LY-294002
*TMEM161B*	−2.339	9.01E-11	-
*VSIG2*	−3.467	9.93E-11	-
*TBCB*	1.255	2.03E-10	-
*CAPN13*	−1.437	2.16E-10	-

^a^ *P* values were calculated using GEO database.

### DMRTA1 May Be Important for GC Development in Male and Female Patients

**Figure 3.**
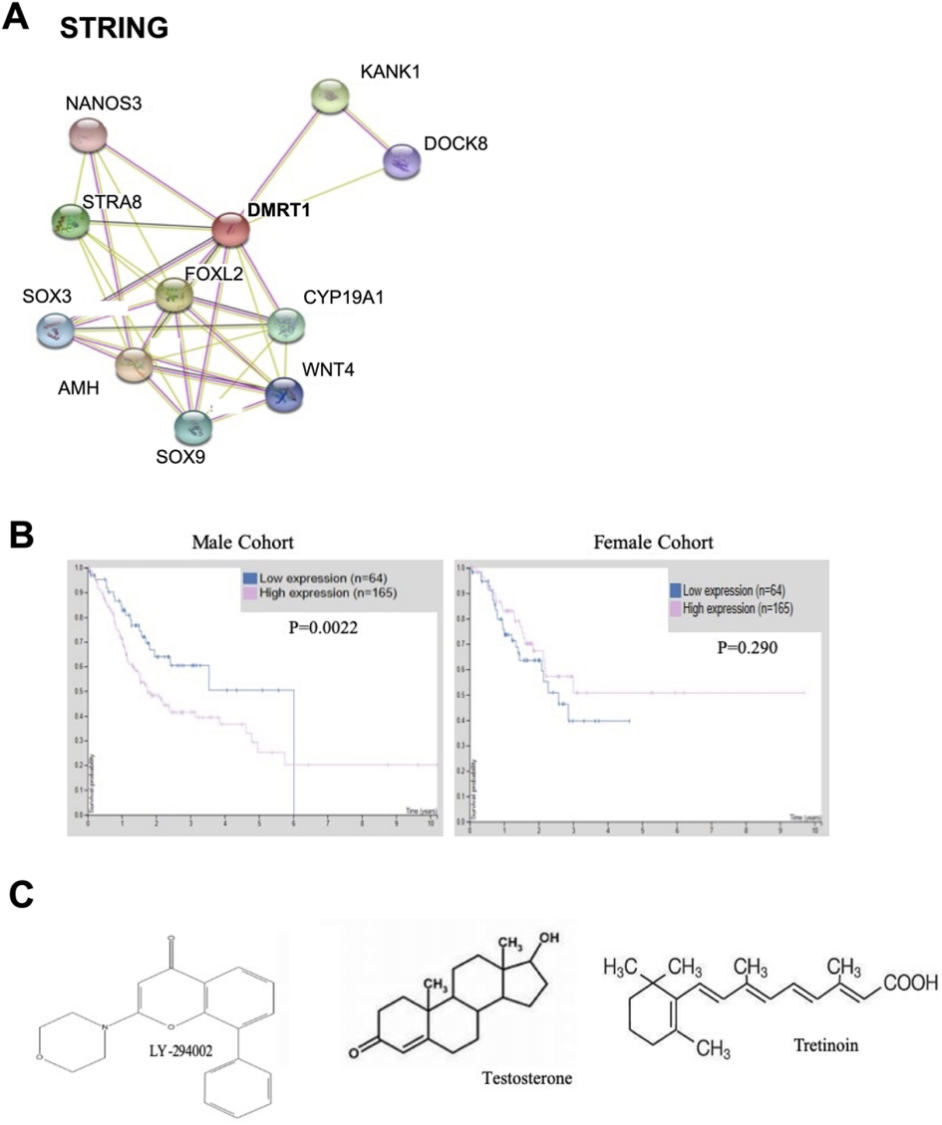
DMRT1 is found to be differentially expressed in male and female patients with gastric cancer. A, STRING database showing DMRT1 protein interactions. B, Survival curves for DMRT1 taken from the human protein atlas for male and female cohorts. C, Drugs that target DMRT1.

We have found a genetic similarity between both gender cohorts with the expression of *DMRTA1. DMRTA1* is a gene normally found to differentiate between the male and female sex in normal cells.^76^ This genetic similarity we have found is interesting because normally *DMRTA1*, when lost in the embryo, leads to female development and when present leads to male development. In not only GC cell lines but in brain-breast metastases, *DMRTA1* was found to be deleted.^77,78^ In an independent publication, *DMRTA1* was also found to be one of the top differentially expressed genes using gene expression data of 50 GC and normal samples.^79^ This observation of differential expression of *DMRTA1* between genders is interesting as its expression pattern is distinctly opposite from the normal genetic functions; female patients have a upregulation whereas male patients have downregulation. Based on these observations, we wanted to understand further the role of *DMRTA1* in patients with GC and the differences within this gene expression between genders. Using the Protein Atlas Database, we have found the male population with low DMRTA1 expression has a significant survival benefit over the high expressers, which correlates with expression found in our male cohort. The female population with high DMRTA1 expression, although not statistically significant, has a slight overall survival benefit over the low DMRTA1 expressers, a trend we observed within our female cohort. The smaller cohort size in the female population may be to blame for the nonstatistical significance (Figure 3A). Due to the presence of this gene in both data sets, we wanted to identify if there were available druggable targets. We utilized the STRING database for protein interaction networks (Figure 3B). *AMH* gene was found to interact with *DMRTA1* and 3 drugs could be utilized to target the protein including LY-294002 (antagonist), testosterone, and tretinoin requiring further investigation (Figure 3C). LY-294002 is an inhibitor of PI3Ks including *AMH* which is also involved in sex differentiation and the cyclic AMP pathway, an interacting pathway of PI3Ks^80^ and has been shown to be biologically active in GC cell lines.^81^ Testosterone depletion is used as a therapy in prostate cancer but has not been explored in GC. Finally, tretinoin is a vitamin A derivative and has been found to have anticancerous effects in GC including targeting the cancer stem cell population.^82^

Stratifying patients with GC based on gender shows distinct molecular differences and highlights more of the vast heterogeneity within GC. It would be logical to infer that because GC affects both men and women, the molecular signatures would be similar for both demographics, but this is not the case. There are clear biological underlying factors within this disease that require further investigation that go deeper than just molecular aberrations. Furthermore, identifying these differences and bringing them to light allows for future discoveries that may impact future GC treatment strategies.

## Conclusion

We have evaluated and compared the molecular landscapes of different subtypes of GC, per the Lauren classification, and between genders. We have found differences in genetic networks between GC and the intestinal (well differentiated), mixed (moderately differentiated), and diffuse (poorly differentiated) cancers. We have also identified differentially expressed genes, which have not been classified earlier in GC. Furthermore, we have noted some genetic diseases occur due to perturbations in the identified genes and may increase the risk of developing GC such as EDS, Marfan syndrome, and hypermethioninemia. We also noted that the mixed subtype of GC might have a genetic component distinctly different from the diffuse subtype while the intestinal subtype lacked any clear evidence of genetic component, which is expected from a pathogenic-induced carcinogenesis. Unfortunately, data sets rarely include messenger RNA sequencing based on the WHO classification while Oncomine only has 1 The Cancer Genome Atlas (TCGA) data set with DNA sequencing available. Furthermore, databases such as TCGA does not stratify based on disease subtype making the analyses more difficult. The existence of various classification systems for GC is ambiguous and if not carefully stated or analyzed within either a preclinical or clinical study, this heterogeneity can influence or skew results. The genetic differences between genders showed vast differences in the top differentially expressed genes. We found a variety of druggable targets that may be effective for female patients that clinically have shown little efficacy in GC. The reason for this is the underrepresentation of females within clinical trials which make identification of an effective therapy difficult. The male patients have more aberrations in tumor suppressive genes and thus finding targeted agents is more difficult. Our group has previously found that selinexor, an inhibitor of nuclear export, effectively retains tumor suppressor proteins and miRNAs within the nucleus and understanding these molecular differences may assist in finding ideal patient populations that would get the most benefit from this therapy or combination therapy. Targeted therapies have shown little efficacy over regular chemotherapies in GC and thus we need to reanalyze the way research is being conducted for this disease. Both researchers and physicians have to collaborate efficiently in order to agree upon the most effective classification system and ways to enhance current GC studies.

## Supplemental Material

Supplemental_Figure_1 - Gastric Cancer Heterogeneity and Clinical Outcomes Click here for additional data file. Supplemental_Figure_1 for Gastric Cancer Heterogeneity and Clinical Outcomes by Rachel E. Sexton, Mohammed Najeeb Al Hallak, Md. Hafiz Uddin, Maria Diab and Asfar S. Azmi in Technology in Cancer Research & Treatment

## Abbreviations

*CLDN1*: claudin 1
EDS: Ehlers-Danlos syndrome
EGFR: estimated glomerular filtration rate
ER: endoplasmic reticulum
5-FU: 5-flurouracil
GC: gastric cancer
miRNA: microRNA
TCA: tricarboxylic acid
TCGA: The Cancer Genome Atlas
TGF-β: transforming growth factor beta
TKIs: tyrosine kinase inhibitors
VEGF: vascular endothelial growth factor
WHO: World Health Organization
