# Supplementary figures and images for: Gastric Cancer Heterogeneity and Clinical Outcomes
Source: Technol Cancer Res Treat. 2020 Aug 17;19:1533033820935477. doi: 10.1177/1533033820935477 (PMC7432987; doi:10.1177/1533033820935477)

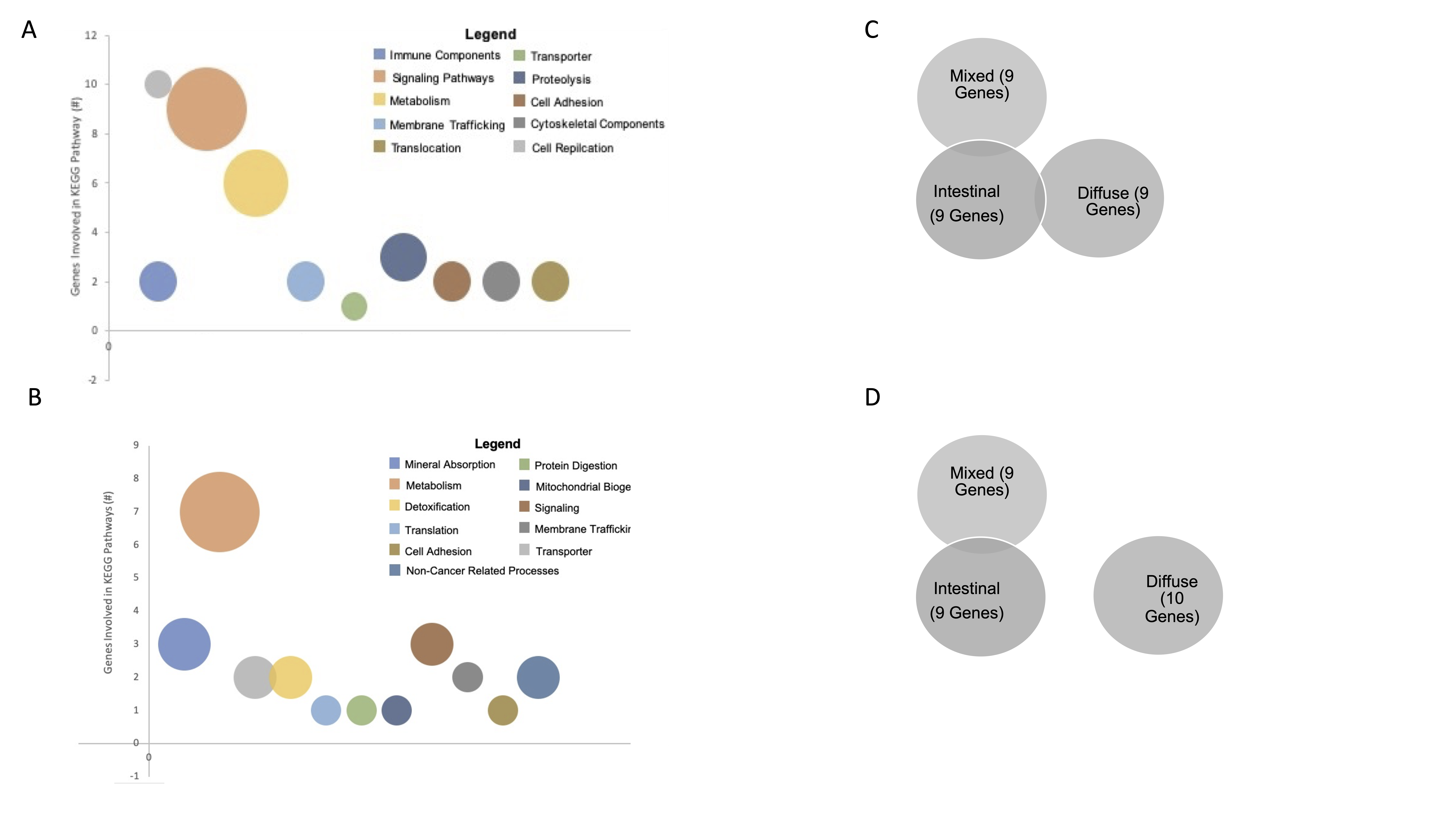

Supplement: Supplemental_Figure_1 - Gastric Cancer Heterogeneity and Clinical Outcomes [file Supplemental_Figure_1.png]
